# How to Tell the Age of a Horse

**Published:** 1884-10

**Authors:** 


How to Tell the Age of a Horse: a pocket manual giving full information of the methods employed by professional horsemen and veterinarians to determine the age of horses, with numerous illustrations showing the shape of the teeth at different ages; and a chapter on horse character, or how to determine the disposition of a horse; with portraits of several famous trotters and thoroughbreds. By Prof. J. M. Heard, M.R.C.V.S., and Professor of Clinical Surgery in the New York College of Veterinary Surgeons. Price, 30 cents. New York: M. T. Richardson, publisher, No. 7 Warren street.

This little book contains a good deal of very valuable information. It is concise, well illustrated, and compact enough to be carried, without trouble, in the pocket. We recommend it to all interested in the horse.

